# Mapping polaronic states and lithiation gradients in individual V_2_O_5_ nanowires

**DOI:** 10.1038/ncomms12022

**Published:** 2016-06-28

**Authors:** Luis R. De Jesus, Gregory A. Horrocks, Yufeng Liang, Abhishek Parija, Cherno Jaye, Linda Wangoh, Jian Wang, Daniel A. Fischer, Louis F. J. Piper, David Prendergast, Sarbajit Banerjee

**Affiliations:** 1 Department of Chemistry, Texas A&M University, Ross@Spence Street, College Station, Texas 77845-3012, USA; 2 Department of Materials Science and Engineering, Texas A&M University, 575 Ross Street, College Station, Texas 77843-3003, USA; 3 The Molecular Foundry, Lawrence Berkeley National Laboratory, Berkeley, California 94720, USA; 4 Material Measurement Laboratory, National Institute of Standards and Technology, Gaithersburg, Maryland 20899, USA; 5 Department of Physics, Applied Physics and Astronomy, Binghamton University, Binghamton, New York 13902, USA; 6 Canadian Light Source, University of Saskatchewan, Saskatoon, Saskatchewan, Canada S7N 2V3

## Abstract

The rapid insertion and extraction of Li ions from a cathode material is imperative for the functioning of a Li-ion battery. In many cathode materials such as LiCoO_2_, lithiation proceeds through solid-solution formation, whereas in other materials such as LiFePO_4_ lithiation/delithiation is accompanied by a phase transition between Li-rich and Li-poor phases. We demonstrate using scanning transmission X-ray microscopy (STXM) that in individual nanowires of layered V_2_O_5_, lithiation gradients observed on Li-ion intercalation arise from electron localization and local structural polarization. Electrons localized on the V_2_O_5_ framework couple to local structural distortions, giving rise to small polarons that serves as a bottleneck for further Li-ion insertion. The stabilization of this polaron impedes equilibration of charge density across the nanowire and gives rise to distinctive domains. The enhancement in charge/discharge rates for this material on nanostructuring can be attributed to circumventing challenges with charge transport from polaron formation.

## Abstract (web summary)

Rapid insertion and extraction of lithium ions from a cathode material is imperative for lithium-ion battery function. Here, the authors present evidence of inhomogeneities in charge localization, local structural distortions and polaron formation induced upon lithiation using scanning transmission X-ray microscopy.

## Graphical abstract

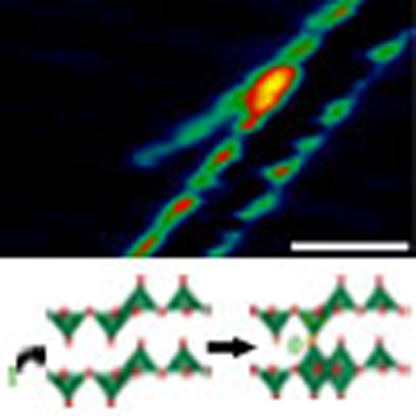

The inadequacies of Li-ion batteries have emerged as a major constraint in many areas of technological design and can be attributed in large measure to challenges with the identification of optimal cathode chemistries and architectures^1^,^2^,^3^,^4^. In essence, a cathode material ought to be able to reversibly store a high concentration of inserted ions and, furthermore, the insertion/extraction and intervening diffusion of ions through the host matrix must occur rapidly to facilitate the efficient discharging/charging of the battery. There are numerous other caveats related to charge transfer at interfaces, earth abundance of the constituent elements and safety considerations that are vital for cathode design. Even this simplified description illustrates the critical imperative to carefully match thermodynamic driving forces of charge transfer (the free energy of the ion insertion reaction) with the kinetics of ion diffusion. In the most ubiquitous example of a Li-ion battery, correlated motion of both ions and electrons must often be considered. These correlations can be driven by the chemical composition, crystal structure and/or electrode geometry of the cathode^1^,^5^,^6^. Electron microscopy and microanalysis probes along with local structure characterization methods, such as total scattering, have provided great insight into the transformation of crystal structures on ion insertion and have enabled identification of numerous bottlenecks, for instance, fracture dynamics, formation of deleterious side products^7^,^8^,^9^,^10^, stabilization of metastable structures or loss of structural homogeneity over repeated charge/discharge cycles. However, the role of electronic structure and its contribution to diffusion barriers for ion migration is less appreciated^5^,^11^. Such diffusion barriers are responsible for the limitations of V_2_O_5_ as a cathode material at high rates and the remarkable (>100,000-fold) enhancement in the performance of this material on nanostructuring^12^,^13^,^14^. Understanding the origin of these diffusion barriers is imperative for developing fundamental design rules for cathode materials to alleviate charge localization.

V_2_O_5_ crystallizes in an orthorhombic layered structure with space group *Pmmn* with a van der Waals' separation of 4.368 Å between the layers (Fig. 1a)^15^,^16^. Three distinct types of oxygen sites can be identified: vanadyl (V=O) oxygen atoms that point between the layers, and bridging and chaining oxygen atoms that connect the polyhedra. V_2_O_5_ was first proposed as a Li-ion intercalation host by Whittingham^18^, owing to the following: the abundance of interlayer sites that can accommodate Li ions; the readily accessible V^5+^/V^4+^ and V^4+^/V^3+^ redox couples; and the strong enthalpic driving forces for Li-ion insertion within this structure^12^,^17^,^18^,^19^. However, despite these promising attributes, the poor high-rate performance of these materials and issues with retention of capacity over prolonged cycling have limited the widespread commercial development of this material. In recent years, this material has enjoyed a resurgence of sorts with the realization that the galleries between V_2_O_5_ layers can accommodate not just Li ions but also other main group and transition metal cations of interest to ‘beyond Li ion' battery chemistries, as well as the understanding that the sluggish kinetics of Li-ion insertion/extraction can be considerably accelerated by scaling to nanometre-sized dimensions^19^,^20^,^21^,^22^.

Electrochemical measurements and spectroscopic probes often reveal the presence of two or more phases when a phase transition accompanies lithiation of a cathode material. In many commercial cathode materials such as LiCoO_2_ or LiMn_2_O_4_, Li insertion occurs with only modest first-order transitions (driven by Li ordering). However, in LiFePO_4_ a pronounced structural transformation between Li-rich and Li-poor phases is involved. Similarly, a number of intercalated phases can be distinguished for Li_*x*_V_2_O_5_ with varying values of *x* depending on the concentration of Li ions inserted within the structure. Figure 1a,b show the structural progression of V_2_O_5_ with increasing intercalation of Li ions; a slightly distorted *α*-phase is initially stabilized for *x<*0.1 and with further lithiation is transformed to the *ɛ*-phase (Fig. 1a), which is stabilized in the range 0.35<*x*< 0.8 with initially cubo-octahedral and then tetrahedral coordination of Li ions; with still more lithiation, a puckered *δ*-phase is stabilized for *ca*. 0.8<*x* < 1.0 (Fig. 1b). In this regime, the phase transitions involve increased separation of the V_2_O_5_ layers, and their puckering and gliding motions to accommodate the structural distortions induced by an increasing concentration of Li ions without requiring cleavage of V–O bonds^1^,^12^,^15^. Further lithiation (*x*>1.0) brings about more pronounced structural distortions that involve bond-breaking and inversion of [VO_5_] polyhedra that are irreversible in the bulk, although there is evidence for recovery of the orthorhombic structure on delithiation for nanostructures^21^,^22^ even for *x* approaching 3 in Li_*x*_V_2_O_5_ (ref. 23). In V_2_O_5_, the phase transitions across the structures depicted in Fig. 1a,b are rapidly accelerated, by up to 100,000 times, on scaling to nanometre-sized dimensions, yet there is evidence from combined Raman and powder diffraction studies of substantial variations in the extents of surface and bulk lithiation^15^,^24^. Understanding the origin of such phase segregation is imperative for the design of cathode materials and geometries.

On chemical lithiation, the ionized Li ion and the electron must diffuse through the solid matrix with the localization of the latter often bringing about a pronounced structural distortion; the combination of the electron and its structural distortion is termed a small polaron, provided the distortion has a length scale comparable to the primitive unit cell of the host material. The signatures of polaron formation and polaron hopping energies have been predicted theoretically, but direct experimental evidence of polaron formation and the accompanying geometric distortions have hitherto not been examined^5^,^25^,^26^,^27^,^28^. Here we present direct evidence of inhomogeneities in charge localization and local structural distortions induced on lithiation using scanning transmission X-ray microscopy (STXM) and corroborate theoretical predictions of a distinctive polaronic state using X-ray absorption near-edge structure and hard-energy X-ray photoemission spectroscopies. The polaron hopping barrier impedes electron diffusion and gives rise to phase inhomogeneity evident as lithiation gradients across an individual particle.

## Results

### Ensemble X-ray absorption measurements of lithiated V_2_O_5_

Chemical lithiation using *n*-butyllithium is used to model Li-ion insertion within a cathode as per the reaction^29^.


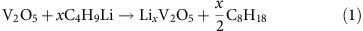


**Figure 1:**
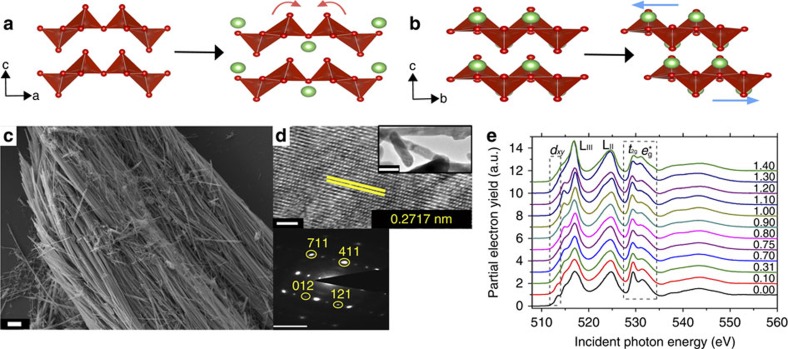
Structural distortions induced on insertion of Li ions and characterization of geometric and electronic structure. As the layered structure of V_2_O_5_ is intercalated with Li ions, it undergoes a series of phase transformations, to a puckered *ɛ*-phase (**a**); on further lithiation, the *ɛ*-phase transforms with an in-plane shift to a *δ*-phase (**b**). (**c**) Scanning electron microscopy images depict V_2_O_5_ nanowires with lengths spanning hundreds of micrometres (scale bar, 3 μm). (**d**) High-resolution transmission electron microscopy (TEM) image of an individual V_2_O_5_ wire (scale bar, 5 nm), indicating the separation between the (711) lattice planes of orthorhombic V_2_O_5_. The top inset shows a low-magnification TEM image of several nanowires (scale bar, 0.2 μm), whereas the bottom inset indicates an indexed selected-area electron diffraction pattern (scale bar, 5 nm^−1^). (**e**) XANES measurements of stoichiometrically lithiated V_2_O_5_ depict a reduction of the 3*d*_*xy*_ resonance at the V L-edge and a diminution of the *t*_2g_ to *e*_g_* ratio at the O K-edge with increasing lithiation.

Figure 1c,d indicate scanning electron microscopy and transmission electron microscopy images of V_2_O_5_ nanowires grown by a previously reported hydrothermal method; the nanowires range from 150 to 250 nm in diameter and span several hundred micrometres in length^15^. The lattice-resolved transmission electron microscopy image in Fig. 1d shows the separation between (711) planes of orthorhombic V_2_O_5_ and, along with the accompanying selected area electron diffraction pattern, indicates that the single-crystalline nanowire grows along the crystallographic *c*-axis direction without any discernible extended defects.

Supplementary Fig. 1 shows an integrated V L- and O K-edge X-ray absorption near edge structure (XANES) spectrum acquired for an individual nanowire of V_2_O_5_ along with putative assignments derived from restricted open-shell configuration interaction with singles quantum chemistry calculations reported by Neese and colleagues^30^ for the V L-edge and our density functional theory (DFT) calculations for the O K-edge, further elaborated below. As an element- and edge-specific probe of unoccupied states, XANES serves as a valuable tool to probe electronic structure and chemical bonding in extended solids and single molecules alike^31^,^32^. The V L-edge is characterized by V L_III_ and V L_II_ spectral features corresponding to transitions from V 2*p*_3/2_→V 3*d* (*ca*. 518 eV) and V 2*p*_1/2_→V 3*d* (*ca*. 525 eV) states, respectively^30^,^33^,^34^, which are split by the spin–orbit coupling of the V 2*p* atomic orbitals of *ca*. 7 eV. In turn, the O K-edge corresponds to transitions from O 1*s* states to states with O 2*p* character. As a result of substantial V 3*d*–O 2*p* orbital hybridization, two distinct sets of resonances are observed, reflecting the crystal field splitting of the V 3*d* orbitals. Assignments of the spectral features illustrated in Supplementary Fig. 1 are derived from DFT modelling and are further corroborated by angle-resolved XANES experiments (wherein the modulation in the intensity of resonances as a function of the polarization reflects the orbital symmetry of the final states)^30^,^33^,^34^.

A Coster–Kronig Auger decay process from a 2*p*_1/2_ into a 2*p*_3/2_ hole renders the V L_II_ feature less informative due to the associated increase in spectral broadening^34^; however, the V L_III_ resonance indicates fine-structure features that strongly depend on the polarization vector; these transitions comprise transitions from the singlet V 2*p*^6^3*d*^0^ into V 2*p*^5^3*d*^1^ states split by crystal field and multiplet effects^30^. Despite convoluting multiplet effects, restricted open-shell configuration interaction with singles calculations^30^ indicate that the first two sharp resonances at 515.6 and 516.8 eV, respectively, correspond to final states that have relatively ‘pure' V 3*d*_*xy*_ and 3*d*_*xz*/*yz*_ character and indeed angle-resolved XANES studies bear out these proposed orbital symmetries^30^,^33^,^34^. In contrast, the O K-edge is not convoluted by multiplet effects and can be clearly distinguished as three sets of transitions from O 1*s* core levels to (a) O 2*p*_*x*_ and 2*p*_*y*_ states that engage in *π* interactions with the *t*_2g_ (V 3*d*_*xz*_, 3*d*_*xy*_ and 3*d*_*yz*_) states of the metal cations (at 529.7 eV); overlapping *σ* states that represent direct end-on hybridization of (b) O 2*p*_*x*_ and 2*p*_*y*_ with V 3*d*_*x*^2^–*y*^2^_ states at 531.6 eV and (c) O 2*p*_*z*_ with V 3*d*_*z*^2^_ states at 533.1 eV. The calculated eXcited-state core hole-X-ray absorption spectroscopy (XCH-XAS) spectrum shown in Supplementary Fig. 1b suggests that the *t*_2g_ manifold is derived primarily from transitions from O 1*s* core levels to O 2*p*_*x*_/*p*_*y*_ states of the vanadyl oxygens that are hybridized with V 3*d*_*xz*_ and 3*d*_*yz*_ states; a lesser contribution to this resonance arises from transitions into O 2*p*_*y*_ states of bridging oxygen atoms hybridized with V 3*d*_*xy*_ states and O 2*p*_*x*_ states of chaining oxygen atoms hybridized with V 3*d*_*xy*_ states. The hybridization of the V=O oxygens with V 3*d*_*xz/yz*_ thus dominates the lineshape of the *t*_2g_ resonance in the O K-edge XANES spectrum with the non-bonding V 3*d*_*xy*_ contributing much less, in contrast to the V L_III_-edge spectrum, wherein the lowest-lying ‘split-off' state is primarily V 3*d*_*xy*_ in origin^30^. Thus, these assignments allow for an orbital-specific description of changes in electronic structure as a function of the lithiation of V_2_O_5_ and provide unprecedented insight into charge localization phenomena.

Figure 1e shows XANES spectra for a series of intercalated V_2_O_5_ samples with increasing values of *x* in Li_*x*_V_2_O_5_. XANES resonances are collected at magic angle (54.7°) incidence to mitigate specific texturation effects^35^. Several trends are immediately discernible (as XANES probes empty orbitals, the diminution of a resonance, to first approximation, corresponds to occupation of states that give rise to the resonance): the first resonance in the V L_III_ spectrum corresponding to transitions to the split-off *d*_*xy*_ conduction band of V_2_O_5_ is strongly diminished with increasing lithiation consistent with the reduction of V_2_O_5_ on lithiation (V^5+^ to V^4+^) and indicating the occupation of the lowest-lying conduction band states. Furthermore, at the O K-edge, the relative intensities of the transitions to the *t*_2g_ and *e*_g_* (π* and σ*) states are greatly modified with the former resonances losing spectral weight. The origin of this pronounced modification of O K-edge spectral lineshapes is distinct from filling of the non-bonding V 3*d*_*xy*_ states and suggests a pronounced rehybridization of V–O bonding at the vanadyl oxygens (*vide infra*).

### Mapping lithiation inhomogeneities within a V_2_O_5_ nanowire

**Figure 2:**
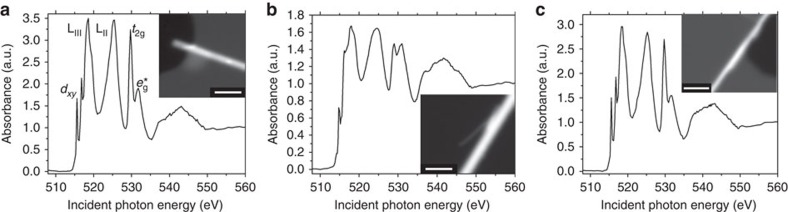
Evaluating electronic structure changes caused by lithium-ion incorporation. STXM image and integrated XANES spectrum acquired for (**a**) an individual V_2_O_5_ nanowire (scale bar, 500 nm), (**b**) an individual nanowire after 1 min of chemical lithiation (scale bar, 200 nm) and (**c**) a lithiated nanowire subjected to delithiation in Br_2_ solution (scale bar, 500 nm). Pronounced differences are discernible after lithiation including diminution of the V L_III_-edge feature attributed to a V 3*d*_*xy*_ final state and the reduction of the *t*_2g_:*e*_g_* ratio. The complete recovery of the electronic structure on delithiation suggests that the spectral changes can be directly attributed to Li-ion intercalation. All spectra have been pre- and post-edge normalized to a unitary absorption cross-section, to depict the relative spectral intensities.

In contrast to ensemble spectra depicted in Fig. 1e, focusing the X-ray beam allows for acquisition of spatially resolved STXM data with *ca*. 25 nm spatial resolution, thereby allowing us to probe the lithiation of an individual nanowire immersed in a toluene solution of *n*-butyllithium for 1 min (Fig. 2b). By finely raster scanning the sample, STXM provides a means to construct a spatially resolved map of the local perturbations to the electronic and geometric structure induced by ion intercalation. Indeed, X-ray imaging has contributed greatly to understanding of inhomogeneities in biomaterials and polymeric systems^36^,^37^. The absolute energy calibration, detector linearity (Supplementary Fig. 2) and beam point spread function are main sources of error for this technique and have been carefully addressed as described in the Methods section^38^.

Figure 2a depicts the STXM image and integrated V L- and O K-edge spectra of an individual V_2_O_5_ nanowire with a diameter of *ca*. 200 nm. In contrast to the orientation-averaged ensemble XANES spectra presented in Fig. 1e, well-resolved lineshapes are discernible for an individual single-crystalline V_2_O_5_ nanowire and the spectral transitions can be assigned as noted in Supplementary Fig. 1 and discussed above. Figure 2b depicts the STXM image and corresponding integrated spectrum acquired for a V_2_O_5_ nanowire after chemical lithiation for 1 min. Pronounced differences are readily discernible in this spectrum; the transition attributed to a V 3*d*_*xy*_ final state at 515.6 eV is greatly diminished in intensity. Concomitantly, at the O K-edge, the *t*_2g_ peaks are broadened and diminished in relative intensity with respect to the *e*_g_* peaks. In concordance with the ensemble XANES spectra (Fig. 1e), the integrated element-specific spectrum in Fig. 2b suggests that the electrons donated by the inserted Li ions have been transferred and reside on the V_2_O_5_ framework but are localized on the lowest-lying V=O 3*d*_*xz/yz*_–O 2*p* hybridized states of the conduction band, which have been substantially distorted as a result of lithiation^17^,^39^,^40^,^41^. Interestingly, on delithiation by immersion in Br_2_ solution the electronic structure of V_2_O_5_ is recovered in its entirety (Fig. 2c), confirming that the electronic structure modulation observed in V_2_O_5_ derives directly from lithiation.

**Figure 3:**
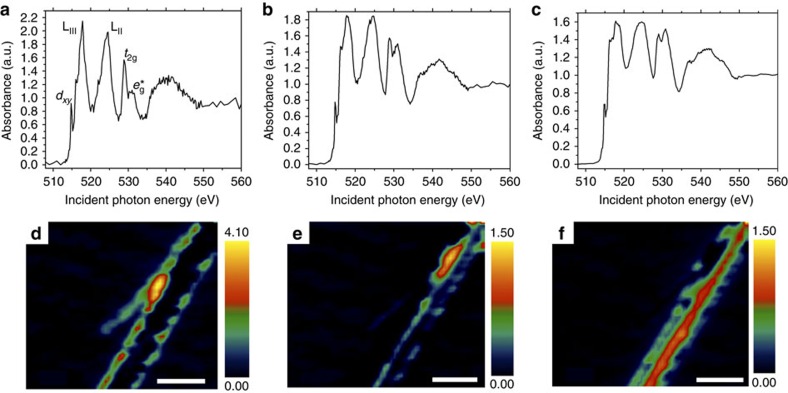
Mapping electron density and inhomogeneous lithiation across a single V_2_O_5_ nanowire. Three distinct spectral contributions deconvoluted from ROI analysis of Fig. 2b are plotted in **a**–**c** in order of increasing lithiation evidenced as a diminution of the V 3*d*_*xy*_ resonance at the V L_III_-edge and the *t*_2g_:*e*_g_* ratio at the O K-edge. Intensity maps for each spectral contribution are plotted in **d**–**f** (scale bar, 200 nm), respectively, showing inhomogeneous regions of lithiation. A nonlinearity correction has been implemented as described by Collins and Ade and described in the Methods section^38^. All spectra have been pre- and post-edge normalized to a unitary absorption cross-section to depict the relative spectral intensities. The colour scale bars represent normalized optical density.

Unlike Fig. 2a–c wherein the spectra show little variation across the span of the nanowires, several distinct spectral contributions are discernible for the lithiated nanowire of Fig. 2b. A region of interest (ROI) analysis allows for identification of three distinct domains that are characterized by spectra individually plotted in Fig. 3a–c; these spectra correspond to different regions of the nanowire shown in Fig. 2b (the spectrum in Fig. 2b captures the integrated spectrum). In going from Fig. 3a–c, the intensity of the *t*_2g_ resonance is progressively diminished with respect to the *e*_g_* resonance, indicating successively greater electronic reduction of the V_2_O_5_ framework; the accompanying maps in Fig. 3d–f indicate the spectral intensities of each of these components across the nanowire, suggesting the presence of distinct domains as a result of inhomogeneous lithiation. These maps are derived based on singular value decomposition of the image stack and by using as a reference the ROI spectra identified within different regions of the same image sequence. This operation produces a set of composition maps where intensities represent the signal strength of each of the spectral components (Fig. 3a–c) in that highlighted area. Notably, Supplementary Fig. 3 shows a thickness map of the nanowire (determined after a nonlinearity correction) along with a cross-sectional scanning electron microscopy image of the surface of an individual nanowire, which indicates that the domains visualized in Fig. 3a–c result from inhomogenous lithiation and do not reflect thickness variations. Figure 3d represents the least lithiated domains within this sample and is weighted most strongly at the periphery of the nanowire; based on the ensemble spectra depicted in Fig. 1e and previous angle-resolved spectra acquired for V_2_O_5_ nanowires^33^, an extent of lithiation in the broad range 0.1<*x<*0.5 can be surmised. It is noteworthy that the intensities at the V L_III_ edge for the lithiated sample are substantially diminished as compared with unlithiated and delithiated V_2_O_5_ as a result of state blocking; occupation of conduction band states diminishes the intensity of the low-energy XANES features. The interiors of the nanowires show two distinct spectral components depicted in Fig. 3e,f with substantially greater extents of lithiation (estimated to be 0.5<*x<*0.9 and 0.9<*x<*1.40, respectively). In particular, Fig. 3f defines a highly reduced strip that runs across a large section of the nanowire. The reduction of the 3*d*_*xy*_ resonance at the V L_III_ edge correlates to the occupation of the lowest-lying levels in the conduction band of V_2_O_5_ by the electron ionized from the inserted Li atom. The resonance observed after lithiation arises from a superposition of remnant V^5+^ and reduced vanadium sites. In contrast, the diminished relative intensity of the low-energy *t*_2g_ peaks at the O K-edge is the result of an induced structural distortion and further polarization of the electron density on V_2_O_5_ caused by the heterogeneous insertion of Li ions (*vide infra*). In other words, the V L_III_ edge allows for direct evaluation of electron density on the vanadium sites, whereas the O K-edge unveils structural distortion of the vanadyl V=O bonds induced as a result of electron localization.

The increased lithiation at the core and the reduced lithiation of the surfaces is explicable based on the orientation of the nanowires (Fig. 1d) and the preferred insertion of Li ions between the layers. In essence, the nanowire is being viewed down the crystallographic *b*-axis and thus is enclosed at the top and bottom by *ab* planes that are impermeable to lithiation. The pronounced differences in lithiation probably further result from the stage ordering typical of layered materials^42^,^43^; the initial stochastic or defect-driven intercalation of Li ions between two specific V_2_O_5_ layers results in a local expansion of the interlayer spacing and facilitates insertion of further Li ions within the same layer. Indeed, Supplementary Fig. 4 indicates a sequence of calculated V_2_O_5_ structures with insertion of one Li ion (Supplementary Fig. 4a) and then two possibilities for insertion of the second Li ion: within the same layer (Supplementary Fig. 4b) and alternating layers (Supplementary Fig. 4c); insertion of Li ions within the same layer is thermodynamically favoured by −0.235 eV per formula unit.

### Structural and electronic distortions induced by lithiation

**Figure 4:**
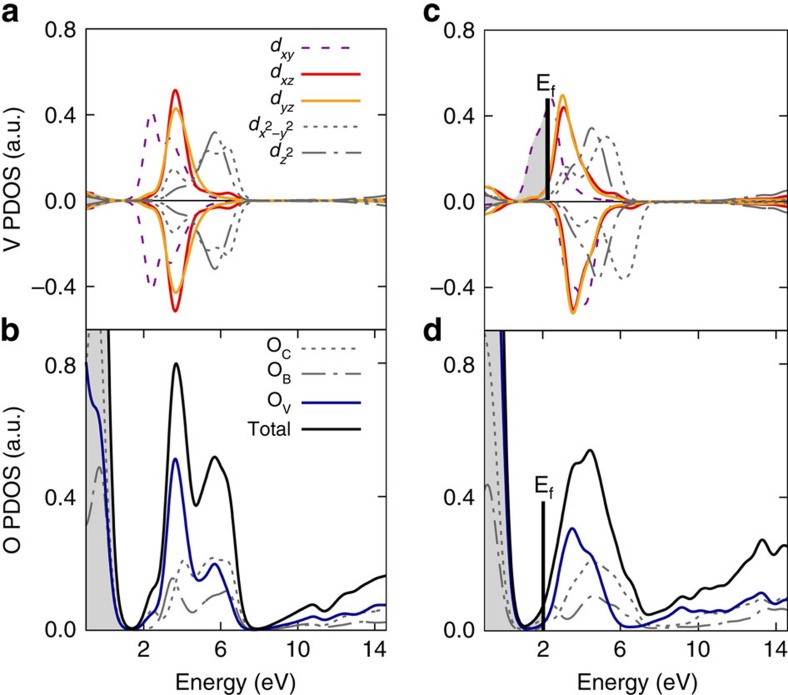
Density of states calculation for V_2_O_5_ and LiV_2_O_5_. The GGA+*U* ground-state pDOS of pristine V_2_O_5_ (**a**,**b**) and the stoichiometric Li_*x*_V_2_O_5_ (**c**,**d**) that adopts the pristine V_2_O_5_ vertical stacking order. The upper panels are the pDOS of vanadium in which the grey area indicates the occupied states. In the lower panels, the key components that are mainly responsible for the changes in the main peak intensity at the O K-edge are outlined by solid curves. The total pDOS (black curves) are the summation of *p*_*x*_-, *p*_*y*_- and *p*_*z*_-components from all three types of oxygen; chaining (O_c_), bridging (O_b_) and vanadyl (O_V_), with the corresponding stoichiometric ratio.

To better understand the variations in the oxygen XANES spectra of the nanowires, DFT+*U* calculations with *U*=3.1 eV^44^, calculations were employed to examine the evolution of the electronic structure as a function of increasing insertion of Li ions^44^,^45^. The on-site Coulomb repulsive energy *U* is essential to capture the effects of strong electron correlation with vanadium 3*d* orbitals. Orthorhombic V_2_O_5_ (Fig. 1a) is a dielectric material with a bandgap of *ca*. 2.3 eV^16^,^46^,^47^,^48^, the conduction band is primarily V 3*d* in character, whereas the valence band has a significant O 2*p* contribution^16^,^30^. The projected density of states (pDOSs) of pristine V_2_O_5_ is shown in Fig. 4a,b, with the valence band maximum aligned at 0. The two spin channels are completely degenerate in this *d*^0^ system with pure V^5+^. In the crystal field of the slightly distorted [VO_5_] square pyramid, the 3*d*_*xy*_ orbitals from the perfectly octahedral *t*_2g_ group are further split into a high-energy component that overlaps with the degenerate 3*d*_*xz*_ and 3*d*_*yz*_ orbital, and a lower-energy component that dominates the conduction band edge^49^,^50^,^51^ (at *ca*. 2 eV above the valence band maximum in Fig. 4a). This lower energy 3*d*_*xy*_ orbital is approximately non-bonding and comprises two split-off bands. The prominent feature in the oxygen total pDOS results primarily from the strong hybridization of the V 3*d*_*xz*_ and 3*d*_*yz*_ orbitals with the vanadyl oxygen atom and indeed these features contribute to the sharp XANES resonance at 529 eV^52^. The secondary feature from the oxygen pDOS is the typical *e*_g_ component and gives rise to the absorption peak at 531 eV. Even without core-hole effects, the ground-state pDOS can still reproduce these features in the V_2_O_5_ O K-edge spectrum (Supplementary Fig. 1b) and enable understanding of the trends observed on lithiation^53^. With increasing lithiation, the donated electrons begin to occupy the non-bonding 3*d*_*xy*_ component at the band edge. We first consider a simple periodic system with a pure V^4.5+^ site, where the electron occupies only a quarter of all available 3*d*_*xy*_ orbitals (as in *α*-NaV_2_O_5_)^51^,^54^. Even at this electron doping level, electron correlation effects are important. The two spin channels are significantly split as a result of the localization of spin states induced by local lattice distortions, which lifts the spin degeneracy as depicted by Fig. 4c. The donated electrons take on 3*d*_*xy*_ character and the localized spins^51^,^54^,^55^ are arrayed along the orthorhombic *b*-axis of V_2_O_5_. Contrary to intuition, the observed diminution of *t*_2g_ intensity is not due to Pauli blocking from electron occupation, as the Fermi level remains far below the main peak position in the quarter-filled case. Instead, the lifted spin degeneracy induced by the correlation effects and lattice distortion plays a much more important role in reducing the *t*_2g_ peak intensity. In short, the oxygen 2*p* components that strongly hybridize with the V 3*d* orbitals are also split into two non-degenerate spin channels, leading to a severe drop in the main peak intensity (Fig. 4d). Further details of the pDOS, illustrating this splitting, are depicted in Supplementary Fig. 5. Both the lifting of spin degeneracy and the lattice distortion caused by lithiation contribute to reduction of the intensity of the *t*_2g_ peak. The inserted Li ions electrostatically attract the vanadyl oxygens towards them and create a pronounced distortion on the *a*–*c* plane (Fig. 1b). Such a distortion further shifts the energetic position of 3*d*_*xz*_ orbitals, resulting in a noticeable migration of *t*_2g_ intensity to higher spectral energies. Based on the abovementioned effects, the pDOS shown in Fig. 4d captures the specific origins of the evolution of the O K-edge spectra.

The Coulomb interaction between the spin-up and spin-down states, represented by the on-site Coulomb repulsion energy *U*, favours the removal of spin degeneracy along one spin polarization so as to lower the total energy of the system (Supplementary Fig. 6) and indeed this stabilization counteracts the elastic energy expended to bring about the distortion of the geometric structure depicted in Supplementary Fig. 7 (also see Supplementary Movie 1). In Supplementary Fig. 6, we initiate a supercell with perfect V_2_O_5_ lattice symmetry and an added electron, and relax the structure by enforcing spin degeneracy. The electron density also becomes delocalized in this case and this delocalized structure is *ca*. 0.22 eV higher in energy than the small polaron structure with a localized electron. These results further suggest that the stabilization of the small polaron in V_2_O_5_ is energetically favoured, both as a result of lattice distortion as well as the lifting of spin degeneracy^56^,^57^. The influence of the Li ion on the small polaron is further discussed below.

To understand the localization of the electron density on Li-ion intercalation, the electronic density difference is calculated from equation (2):


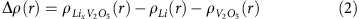


where 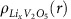 is the electron density of the Li-intercalated V_2_O_5_, 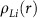 of isolated Li atoms in the same position as in the total system and 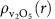 for V_2_O_5_. Supplementary Fig. 4 plots the increase and decrease in electron density of singly lithiated Li_0.125_V_2_O_5_ and doubly lithiated Li_0.25_V_2_O_5_ systems. The increase in electron density traces the contours of a V 3*d*_*xy*_ orbital, indicating an electron localized in this lowest-lying state of the conduction band; in contrast, the electron density decrease is localized within bonds along the [VO_5_] pyramid. To put it differently, the increased electron density localized on the V 3*d*_xy_ orbitals polarizes the V–O bonds and brings about a pronounced increase of the bond length. The coupled charge localization and distortion of the geometric structure further defines a small polaron as observed in the single-electron reduction case. Supplementary Movie 1 illustrates the localized distortion of the structure wherein the V atom shifts away from the intercalated Li ion; the bridge and chain oxygen atoms distort away from the central vanadium atom reflecting increased bond lengths and the vanadyl oxygen atoms orient towards the intercalated Li ions defining its cubo-octahedral local coordination environment.

### Stabilization of a small polaron

Polaronic confinement in transition-metal oxides has been extensively examined using DFT calculations^5^,^25^,^26^. Ioffe and Patrina^27^ have previously attempted to correlate the conductivity of V_2_O_5_ to small polaron formation using electronic transport measurements. However, direct atomic-scale evidence of polaron formation has thus far been elusive. The clear correlation of transitions related to the final states involving the V 3*d*_*xy*_ level on lithiation noted in Fig. 2 and the subsequent effect on the crystal structure of the material shown in the reduction of the *t*_2g_/*e*_g_* ratio clearly indicates that polaron formation plays a key role in limiting Li diffusion within this material.

The energetic barrier to polaron diffusion within Li_0.125_V_2_O_5_ was calculated. Previous studies have shown strongly disfavoured Li migration along the *a* and *c* axes with migration barriers of 1.88 and 1.69 eV, respectively^58^. Supplementary Fig. 8a,b show a schematic depiction of the path adopted by Li ions between adjacent Li sites along the *b* axis, with a calculated diffusion barrier of 0.22 eV^19^. The diffusion of Li ions involves a change in the local coordination environment from 8→3→8 anions. Supplementary Fig. 8c illustrates the constrained trigonal planar transition state; the energetic barrier derives in large part from the substantial change of coordination number and the unfavourable coordination environment in the transition state. To further understand electron–polaron interactions with the intercalated Li ion, the polaron formation energies are calculated for two separate pairs of vanadium positions (Supplementary Fig. 9). In the first case, the electron is localized on the V1–V2 pair (in the proximity of the Li ion), whereas in the second case the electron is localized on the V3–V4 pair (far from the Li ion). The former configuration yields a formation energy of −0.41 eV/V_2_O_5_ unit, suggesting that the polaron is stabilized by an attractive interaction with the Li ion. In contrast, the calculated formation energy for the latter configuration with the polaron situated at V3–V4 positions is 0.02 eV/V_2_O_5_, clearly a much less stable configuration for the polaron. Supplementary Fig. 9c indicates that the migration barrier for the V1–V2 polaron is 0.34 eV, whereas the comparable value for the V3–V4 pair is 0.03 eV. These calculations thus suggest that small polarons are preferentially stabilized adjacent to the intercalated Li ions but this stabilization also entails a substantial barrier for migration of the polarons along the V_2_O_5_ framework^56^. In other words, the intercalated Li ions play a critical role in stabilizing the polaron and determining its ease of migration.

**Figure 5:**
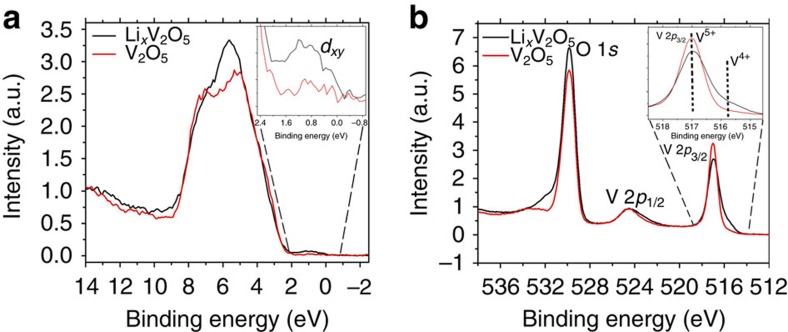
Valence band and HAXPES measurements of V_2_O_5_ and Li_*x*_V_2_O_5_. (**a**) Valence band spectra for pristine V_2_O_5_ (red) and Li_*x*_V_2_O_5_ (black). The right inset depicts a magnification of the region showing the emergence of a feature below the Fermi level. As predicted by theory, HAXPES measurements clearly illustrate the appearance of a polaronic state below the Fermi level. (**b**) HAXPES performed on these samples demonstrates the existence of V^4+^ and V^5+^ sites at the V 2*p*_3/2_ peak.

Now, turning our attention to the electronic consequences of Li-ion intercalation, the calculated orbital pDOSs in Supplementary Fig. 10 suggest that lithiation should be accompanied by the appearance of a ‘mid-gap' state between the valence and conduction band. To examine the predictions of the appearance of a filled state derived from polaron formation in the upper valence band, hard X-ray photoemission spectroscopy (HAXPES) measurements have been performed for lithiated samples (Fig. 5). The V 2*p*_3/2_ spectrum in Fig. 5b clearly indicates the presence of discrete V^4+^ and V^5+^ states. Most notably, the inset to Fig. 5a indicates the emergence of a feature not observed for orthorhombic V_2_O_5_ at *ca*. 1.0 eV below the Fermi level in the valence band spectrum that corroborates and serves as a distinctive signature of the mid-gap polaronic state predicted by DFT. The appearance of this state provides definitive experimental evidence for localized electrons corresponding to stabilization of a small polaron.

## Discussion

The experimental results in concert with the calculations indicate that local structural distortions and the stabilization of small polarons impede electron diffusion within V_2_O_5_ and give rise to distinctive lithiation gradients^42^,^59^. The STXM images correspond to a map of electron density on the V_2_O_5_ framework, which further reflects the lithiation gradient as a result of the close association of localized electrons with Li ions. As noted above, in Li_*x*_V_2_O_5_, the Li-ion stoichiometry *x* determines the phase of the material and a series of phase transformations are evidenced with increasing lithiation. Barriers to diffusion of Li ions thereby also influence the sequence of structural phase transformations. In other words, STXM provides a view of trapped electron density, which is correlated to lithiation gradients, further reflecting barriers to propagation of phase transformation within an individual Li_*x*_V_2_O_5_ nanowire. The pronounced increase in high-rate performance as observed for nanostructures thus probably results in a much more facile phase nucleation enabled by easier electron and ion diffusion.

In summary, we have mapped the changes in electronic structure and local structural distortions induced by the lithiation of V_2_O_5_ using a combination of V L-edge and O K-edge XANES and STXM probes of the conduction band and HAXPES examination of the valence band; the spectra are interpreted with the assistance of DFT+*U* calculations. Specifically, we note the stabilization of distinctive domains within individual nanowires of lithiated V_2_O_5_ corresponding to the emergence of charge density gradients along the nanowires that can be correlated to inhomogeneous lithiation. These measurements provide the first view of highly anisotropic lithiation of layered materials resulting from the peculiarities of their electronic and geometric structure. Spectral assignments verified by DFT calculations suggest that lithiation of V_2_O_5_ induces the localized reduction of specific vanadium sites with the electron derived from the ionized Li ion residing in neighbouring V 3*d*_*xy*_ orbitals that are the lowest-lying states in the conduction band. In a complementary manner, O K-edge XANES spectra and STXM maps depict the local structural distortions induced by exchange interactions and small polaron formation as a result of strong modification of V–O hybridization along the vanadyl V=O bonds. DFT calculations confirm that electron density localization is sufficient to drive elastic distortion of the local atomic structure. The quasiparticle comprising the trapped electron and the local distortion constitutes a small polaron and polaronic signatures predicted by DFT have been verified by HAXPES studies. Delithiation of V_2_O_5_ brings about elimination of the polaron and complete recovery of the electronic structure. The small polaron formation directly evidenced in these studies is thought to be the origin of sluggish diffusion of Li ions through the cathode, with a diffusion barrier of *ca*. 0.22 eV, limiting high-rate performance. The strongly accelerated kinetics of lithiation observed on scaling to nanometre-sized dimensions can also, in large measure, be attributed to the ability to circumvent the limitations of sluggish small polaron hopping at these sizes. The fundamental limitations to ion diffusion unveiled here suggest that V_2_O_5_ cathode materials will benefit from development of quasi-amorphous or highly porous materials where charge is not required to travel large distances or by devising novel lattice frameworks with lower extent of polaronic confinement. Quasi-amorphous or highly porous materials would have a greater contact area with the electrolyte, thereby greatly limiting the range over which small polaron hopping needs to be sustained and mitigating the kinetic impediments imposed by stabilization of a polaron.

## Methods

### Synthesis and chemical lithiation of V_2_O_5_ nanowires

Synthesis and the subsequent lithiation of the V_2_O_5_ nanowires were carried out as previously reported^15^. Briefly, V_2_O_5_ nanowires were synthesized via hydrothermal reduction of bulk V_2_O_5_ (Sigma-Aldrich, 99.5%) with oxalic acid (J.T. Baker), to prepare V_3_O_7_·H_2_O nanowires, followed by oxidation in air at 300 °C to obtain phase-pure V_2_O_5_ nanowires. Lithiation was carried out within a glove bag under Ar ambient via immersion of the powder in molar excess (4:1 Li:V_2_O_5_) of 2.5 M *n*-butyllithium solution in hexanes (Sigma-Aldrich) diluted to 0.025 M in toluene. Delithiation was accomplished by immersion of the lithiated samples in pure liquid Br_2_ for 2 h, followed by washing with large amounts of hexanes. The samples are sealed within a glovebox for transport to synchrotrons for XANES and STXM measurements.

### XANES spectroscopy

XANES measurements were carried out at the National Synchrotron Light Source of Brookhaven National Laboratory at beamline U7A operated by the National Institute of Standards and Technology with a toroidal mirror spherical grating monochromator using a 1,200 lines per mm grating with a nominal energy resolution of 0.25 eV with a slit size of 30 × 30 μm. XANES spectra were collected in partial electron yield mode with a channeltron multiplier near the sample surface; the detector was used with an entrance grid bias of −200 V bias to reject low-energy electrons; a charge compensation gun was used to avert the charging of the samples. The incident beam is linearly polarized 85% in the plane of the synchrotron ring. As XANES uses linearly polarized light and incorporates dipolar transitions, the absorption cross-section transforms as follows:


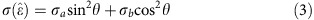


where 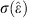 is the Cartesian tensor for the absorption cross-section derived from Fermi's Golden rule, *σ*_*a*_ and *σ*_*b*_ are distribution functions of crystallite orientation and *θ* is the angle between the polarization vector and sample. If *θ*=54.7°, the isotropic average is:


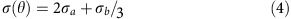


and thus specific texturation effects are heavily mitigated at this angle.

The partial electron yield signals were normalized using the incident beam intensity, to eliminate the effect of incident beam intensity fluctuations and monochromatic absorption features. The V L- and O K-edge spectra were acquired in a single scan. Data were collected along a metallic vanadium reference mesh for energy calibration. Pre- and post-edge normalization of the spectrum was performed using the Athena suite of programmes.

### Scanning transmission X-ray microscopy

STXM measurements were performed at the SM (10-ID1) beamline of the Canadian Light Source, a 2.9-GeV third-generation synchrotron facility. A 25-nm outermost-zone zone plate was used to obtain a diffraction-limited spatial resolution better than 30 nm. A 500-line per mm plane grating monochromator was used to acquire the V L-edge and O K-edge spectral stacks. The incident photon flux (*I*_o_) count rate was adjusted to be <20 MHz and optimized to *ca*. 17 MHz as read by the STXM detector within a hole located close to the sample of interest and measured at 560 eV by adjusting the exit slits to 17/16 μm (dispersive/non-dispersive). The V L- and the O K-edge stacks covered an energy range from 508 to 560 eV with energy steps of 0.2 eV in the ROI and 1 eV in the continuum region beyond the specific elemental edges with dwell time of 1 ms for each section. Right circularly polarized X-rays, generated by an elliptically polarized undulator was used in the experiments. All STXM data were analysed and processed using aXis2000 software (http://unicorn.mcmaster.ca/aXis2000.html). STXM maps are derived based on singular value decomposition of the image stack in aXis2000 and by using as a reference the ROI spectra identified within different regions of the same image sequence. This operation produces a set of composition maps where intensities represent the signal strength of each of the spectral components (Fig. 3a–c) at each specific pixel of that highlighted area. To correct for the nonlinearity of the detector, the flux was measured as a function of dispersive slit width for non-dispersive slit widths of 5, 10, 15 and 25 μm at 560 eV (Supplementary Fig. 2). The resultant curves were fit using the function as per the method described by Collins and Ade^38^:


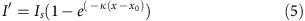


where *I*′ is the measured flux, *I*_s_ is the detector saturation flux, *κ* is the rate at which measured flux approaches saturation, *x* is the dispersive slit width and *x*_0_ is a slit width zero offset. The parameters extracted from the fit function allow for the measured flux to be corrected to the actual flux using the following relationship:


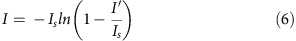


From this analysis, the quantum efficiency of the detector can be determined as a function of flux and is plotted in Supplementary Fig. 2 (ref. 38). The experimental spectrum of the lithiated V_2_O_5_ nanowires was corrected by first extracting the average measured flux at each pixel from the image stack. The extracted flux was then corrected by calculating the actual flux, as in latter equation, yielding a spectrum representative of the actual flux values at each pixel. Owing to the magnitude of correction being dependent on the measured flux, a correction factor was then calculated for each pixel, which was then multiplied by the stack to yield a corrected image (Fig. 3).

### Hard X-ray photoemission spectroscopy

HAXPES measurements were performed at the National Institute of Standards and Technology bending magnet beamline X24 of the National Synchrotron Light Source of Brookhaven National Laboratory. Measurements were performed at a *ca*. 4 keV photon energy with a pass energy of 500 eV and a Gaussian instrumental broadening of 0.45 eV. The higher excitation of HAXPES circumvents serious charging issues that are common to ultraviolet and soft X-ray photoelectron spectroscopy. No evidence of charging was observed during our measurements. The HAXPES spectra are energy aligned to the Fermi level of a gold foil reference in electrical contact with our samples, unless stated otherwise. To mitigate further energy alignment shifts from beam drift, the Au reference scans were measured before and after each spectrum.

### Computational details

The ground-state structural and electronic properties of V_2_O_5_ and the lithiated systems are obtained using DFT^60^,^61^ with the Vienna *ab initio* simulation package^62^. The exchange-correlation energies are calculated within the specific generalized-gradient approximation (GGA) of Perdew–Burke–Ernzerhof 63. The electron–ion interaction is treated with projector-augmented-wave pseudopotentials^64^,^65^, using a 400-eV plane-wave kinetic energy cutoff. A rotationally invariant DFT+*U* approach^45^ is employed to describe the on-site Coulomb interaction of the spin-up and spin-down electrons, with *U*=3.1 eV^44^. To converge the total energy, we sample the first Brillouin zone with a Monkhorst–Pack reciprocal space grid of 6 × 6 × 6 **k**-points. All the atomic structures being considered have been relaxed until each Cartesian force component is no greater than 0.01 eV Å^−1^. To guarantee highly resolved pDOS, we calculate Kohn–Sham eigen energies based on the converged electron density on a grid of 24 × 24 × 24 **k**-points centred at the zone centre (**Γ**-point). The pDOS is numerically broadened with a Fermi-Dirac smearing of 0.2 eV, approximately mimicking the intrinsic broadening due to the oxygen 1*s* core-hole lifetime. Higher-resolution pDOS shown in the Supplementary Materials is obtained from the much smaller broadening of 0.03 eV. The Tkatchenko–Scheffler method was used to describe the van der Waals interaction between the layers of V_2_O_5_ (ref. 66). Lithium-ion diffusion barriers in *α*-Li_0.125_V_2_O_5_ are calculated using the nudged-elastic band (NEB) method^67^ as implemented in the Vienna *ab initio* simulation package. A total of seven images are calculated between the end points to capture the energy landscape for Li ion diffusion. The end points are optimized to a force tolerance of ±0.001 eV Å^−1^, whereas the convergence criterion for the forces along the NEB path is 0.1 eV Å^−1^ (ref. 68).

### Data availability

The data that support the findings of this study are available from the corresponding authors (DP and SB) upon request.

## Additional information

**How to cite this article:** De Jesus, L. R. *et al.* Mapping polaronic states and lithiation gradients in individual V_2_O_5_ nanowires. *Nat. Commun.* 7:12022 doi: 10.1038/ncomms12022 (2016).

## Supplementary Material

Supplementary Information Supplementary Figures 1-10 and Supplementary References.

Supplementary Movie 1 Structural Distortion Induced by Lithium Intercalation. Transformation of a single unit cell of V_2_O_5_ is characterized by the puckering of the apical oxygen towards the Li-ion, while the vanadium atoms rearrange by moving away. This distortion causes the localization of electron on the vanadium atom, creating a small polaron.
