# Supplementary material for: Mapping polaronic states and lithiation gradients in individual V2O5 nanowires
Source: Nat Commun. 2016 Jun 28;7:12022. doi: 10.1038/ncomms12022 (PMC5411759; doi:10.1038/ncomms12022)
Supplement: Supplementary Information — Supplementary Figures 1-10 and Supplementary References. [file ncomms12022-s1.pdf]

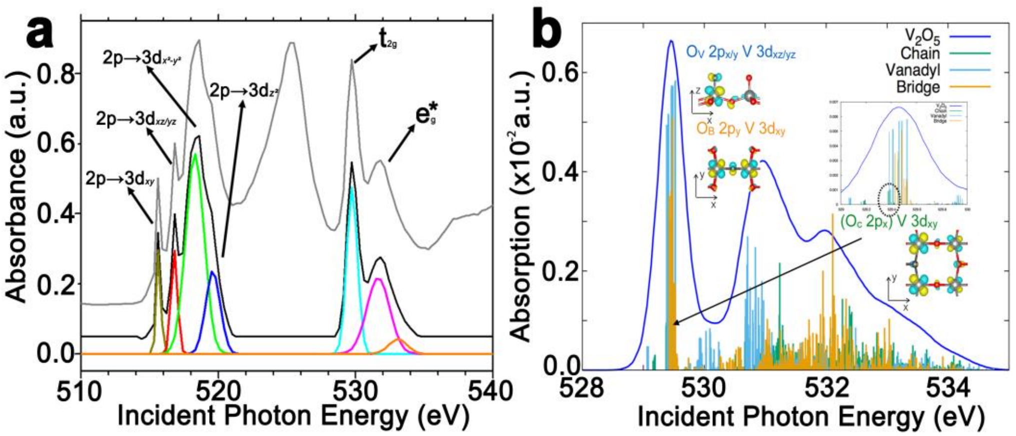

Supplementary Figure 1. Peak assignments for V L- and O K-edge X-ray absorption near edge structure (XANES) Spectra (a) Assignments of V L_{III}-edge spectral features to specific transitions measured for an individual V_2O_5 nanowire as calculated by Manganas *et al*.[1] (b) *Ab-initio* calculated spectrum for V_2O_5 depicting major contributors for the t_{2g} and e_g* peaks at the O K-edge.

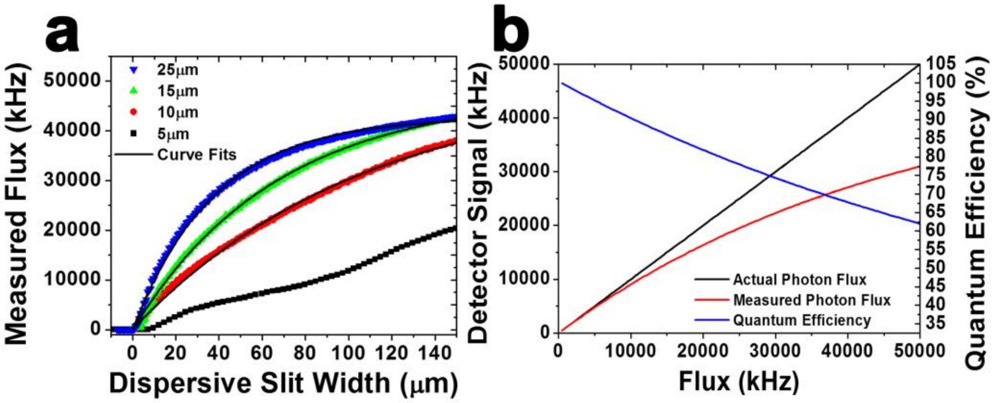

Supplementary Figure 2. Corrections for Detector Nonlinearity (a) Detector response as a function of dispersive slit widths for specified non-dispersive slit widths (5, 10, 15, and 25 μm). The latter three plots have been fitted by the function indicated as Equation 5 of the Methods section. The fits are indicated by solid lines. (b) Measured flux and quantum efficiency as a function of actual photon flux for a non-dispersive slit width of 25 μm.

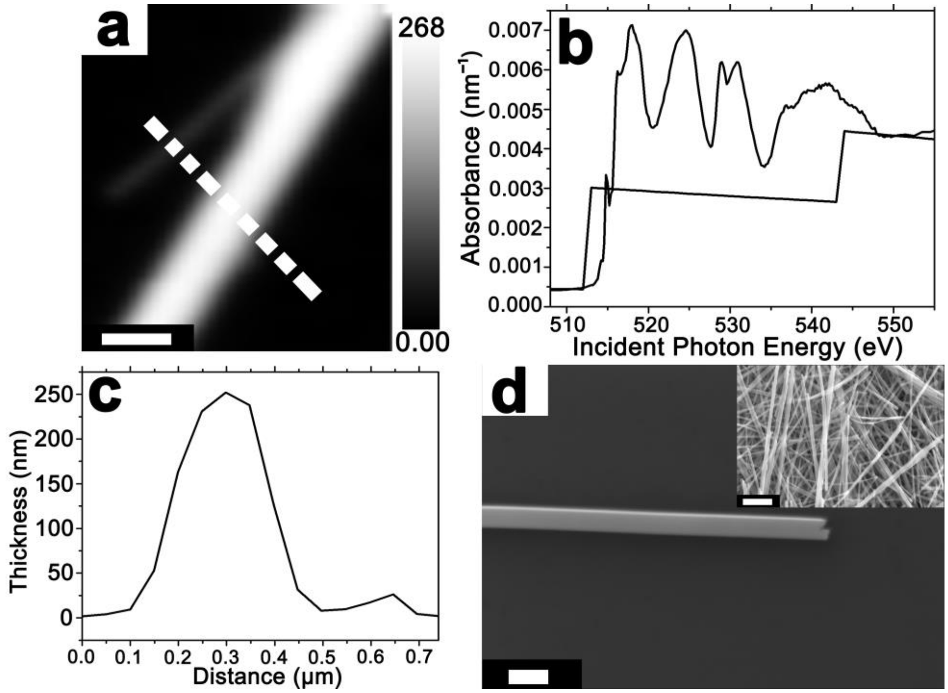

Supplementary Figure 3. V L- and O K-edge chemical imaging of a single lithiated V_2O_5 nanowire Thickness map (a) for a 1 min lithiated V_2O_5 nanowire where the gray scale represents the thickness in nm (scale bar, 200 nm), the dashed line represents the section surveyed in (c). Linearly scaled reference spectrum for $Li_xV_2O_5$ (b) used to construct the thickness map. High-resolution scanning electron microscopy images of a single V_2O_5 nanowire depicting a uniform rectangular cross-section (d) (scale bar, 200 nm).

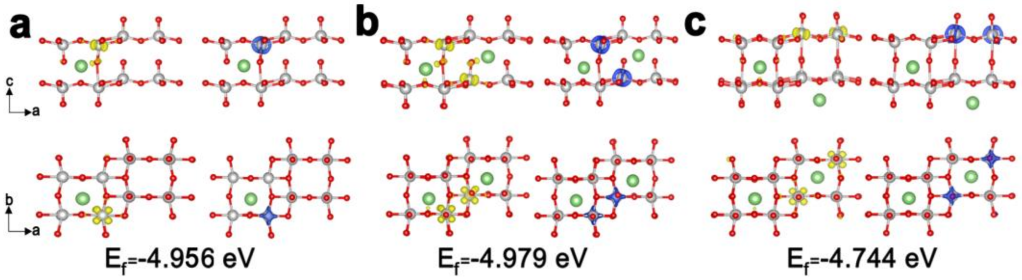

Supplementary Figure 4. Stage Ordering of Li-ion Insertion Charge density differences calculated for V_2O_5 with one intercalated Li-ion (a), two Li-ions incorporated within the same layer (b), and two Li-ions incorporated in alternate layers (c). The increase of charge density is depicted in yellow and decrease of charge density is depicted in blue. The increased charge density is observed to trace the contours of the V $3d_{xy}$ orbital. The decrease in charge density is localized between the bonds showing polarization of the bonds between V–O bonding. The calculated formation energies depict a preference for successive lithiation of the same layer, as compared to alternate layers.

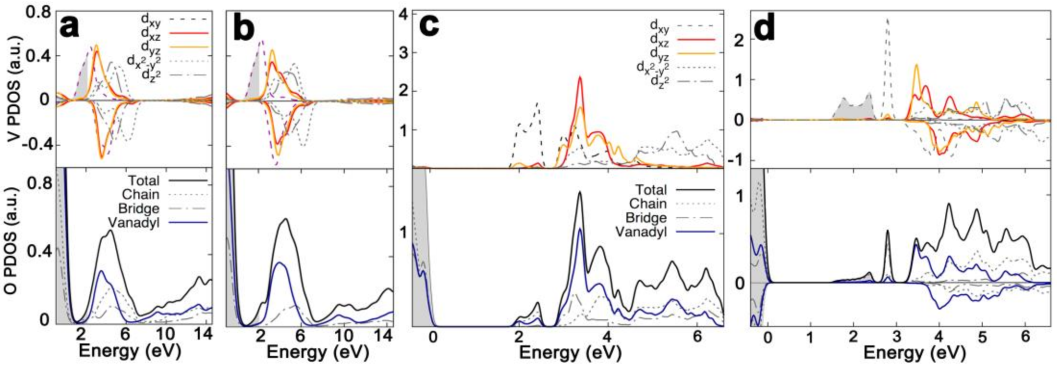

Supplementary Figure 5. **Projected density of states (pDOS) for (a) α-LiV_2O_5 and (b) δ-LiV_2O_5** Supplementary Figure 5 demonstrates the impact of stacking order on the pDOS. It can be seen that in both cases, the spin-up and –down channel are split much in the same manner, except that the d_{xz}-component (red curves) is shifted to higher energies in going from (a) α-LiV_2O_5 to (b) δ-LiV_2O_5. This is because the relative gliding of the V_2O_5 layers in δ-LiV_2O_5 causes the inserted Li-ion to situate at the mid-point of two dangling vanadyl oxygens, which substantially magnifies the lattice distortion on the *ac* plane. pDOS broadened with 0.03 eV for (c) V_2O_5 and (d) α-LiV_2O_5. This data directly corresponds to the calculated Fig. 4. Two spin-components are shown for α-LiV_2O_5. The DFT+*U* calculation predicts that the lifting of spin degeneracy causes the spin-up and –down components of 3d states to be split by ca. 0.7 eV. The large spin-channel splitting is also evident in the oxygen 2p states, which is the primary cause for the diminution of the sharp t_{2g} resonance in the O K-edge absorption spectra.

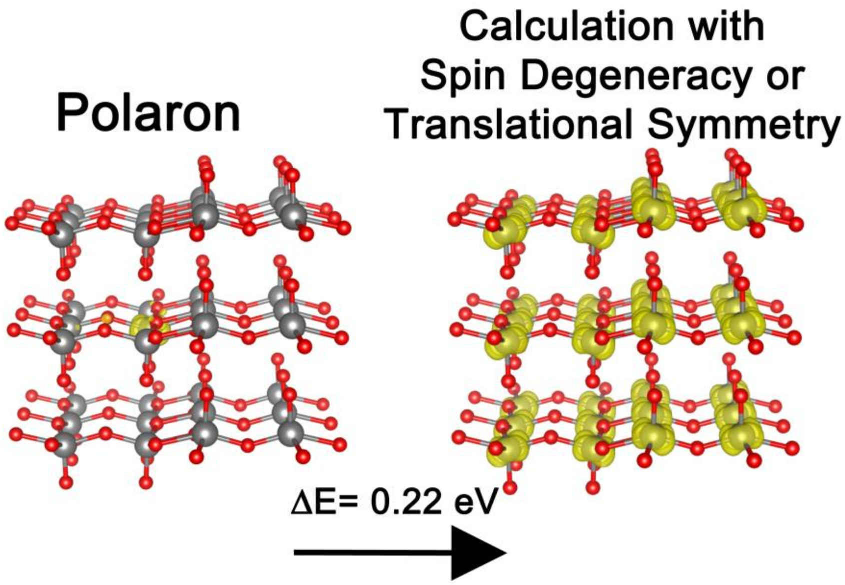

Supplementary Figure 6. Charge localization upon addition of a single electron in pristine V_2O_5 The polaron formation in the V_2O_5 structure is dependent of the electron spin. Enforcing spin-degeneracy on a supercell of V_2O_5 results in charge delocalization throughout V_2O_5 and a total energy increase of roughly 0.22 eV for the supercell with 24 V atoms. This suggests the polaron is energetically favorable due to correlation effects and is stabilized *via* symmetry breaking mechanisms.

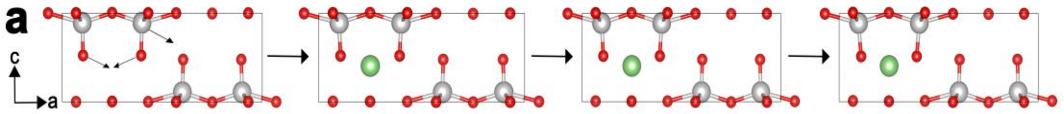

Supplementary Figure 7. Structural Distortion Induced by Polaronic Confinement A schematic depiction of the transformation of a single unit cell of V_2O_5 upon intercalation of a Li-ion.[2,3] A sequence of structural changes is depicted with puckering of the apical oxygen towards the lithium ion and rearrangement of the vanadium ions away from the intercalated Li-ion. Supplementary Movie 1 illustrates the distortions induced in V_2O_5 upon lithiation.

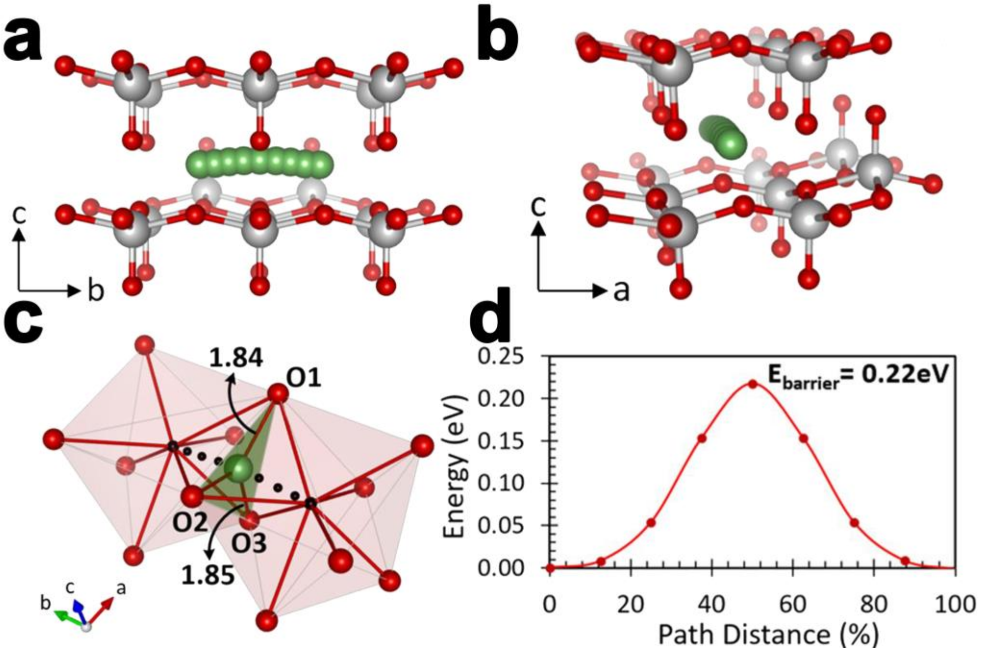

Supplementary Figure 8. Nudged elastic band studies of Li-ion Diffusion Pathways Schematic depicting the migration pathway adopted by a Li-ion along the *b*-axis; as observed along the *a*-axis (a) and the *b*-axis (b). A view of the oxygen coordination environment at the transition state in α-V_2O_5 (c). The minimum diffusion pathway energy is calculated to be 0.22 eV for a Li-ion moving along the *b*-axis of α-$Li_xV_2O_5$ (d).

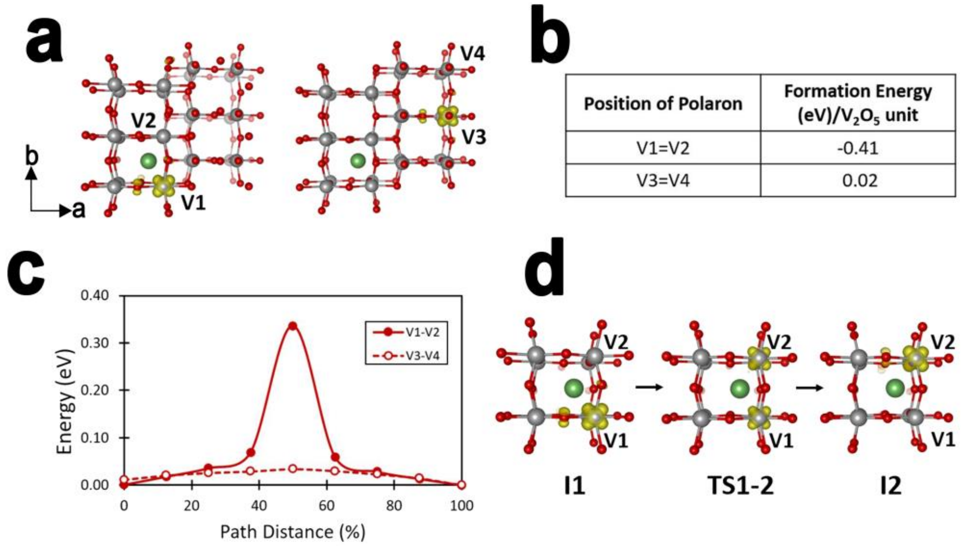

Position of Polaron	Formation Energy (eV)/V_2O_5 unit
V1=V2	-0.41
V3=V4	0.02

Supplementary Figure 9. Nudged elastic band studies of V^{4+} polaron diffusion pathways (a) The formation of a polaron at position V1 (or V2) close to the Li-ion or positions V3 and V4 relatively far from the Li-ion. (b) Tabulated values of formation energies for the two configurations depicted in (a). (c) Calculated migration barriers for hopping of polarons situated at (V1, V2) sites in proximity of the Li-ion (solid line) and relatively far away from the Li-ion (V3, V4) (dashed lines). The steps involved in polaron diffusion are depicted in (d) All the orbitals are plotted at a value of electron density equal to 0.015 e$Å^{-3}$.

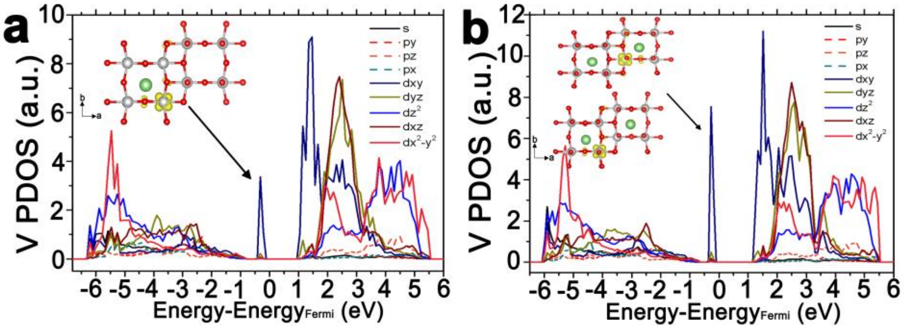

Supplementary Figure 10. Orbital-projected density of states for V_2O_5 with one- or two- inserted Li-ions A “mid-gap” state is observed to appear within the bandgap as a result of lithiation and is experimentally corroborated by the HAXPES results shown in Figure 5. (a) Orbital-projected density of states for one Li-ion and (b) two Li-ions incorporated per V_2O_5 unit cell. The insets depict the charge density of the mid-gap states.

Supplementary References:

1. Maganas, D. *et al.* First principles calculations of the structure and V L-edge X-ray absorption spectra of V_2O_5 using local pair natural orbital coupled cluster theory and spin-orbit coupled configuration interaction approaches. *Phys. Chem. Chem. Phys.* **15,** 7260–76 (2013).

2. Perez-Mato, J. M., Orobengoa, D. & Aroyo, M. I. Mode crystallography of distorted structures. *Acta Crystallogr. Sect. A* **66,** 558–590 (2010).

3. Orobengoa, D., Capillas, C., Aroyo, M. I. & Perez-Mato, J. M. AMPLIMODES: symmetry-mode analysis on the Bilbao Crystallographic Server. *J. Appl. Crystallogr.* **42,** 820–833 (2009).
